# Harmonisation of assessments of attention, social, emotional, and behaviour problems using the Child Behavior Checklist and the Strengths and Difficulties Questionnaire

**DOI:** 10.1002/mpr.2001

**Published:** 2024-01-12

**Authors:** Nicole Baumann, Peter J. Anderson, Samantha Johnson, Neil Marlow, Dieter Wolke, Julia Jaekel

**Affiliations:** ^1^ Department of Population Health Sciences University of Leicester Leicester UK; ^2^ Turner Institute for Brain & Mental Health School of Psychological Sciences Monash University Melbourne Victoria Australia; ^3^ Department of Psychology University of Warwick Coventry UK; ^4^ Clinical Sciences Murdoch Children's Research Institute Melbourne Victoria Australia; ^5^ UCL Elizabeth Garrett Anderson Institute for Women's Health University College London London UK; ^6^ Warwick Medical School University of Warwick Coventry UK; ^7^ Psychology University of Oulu Oulu Finland; ^8^ Public Health Unit Finnish Institute for Health and Welfare (THL) Helsinki Finland

**Keywords:** CBCL, data harmonisation, measurement invariance, mental health, SDQ

## Abstract

**Objectives:**

Retrospective harmonisation of data obtained through different instruments creates measurement error, even if the underlying concepts are assumed the same. We tested a novel method for item‐level data harmonisation of two widely used instruments that measure emotional and behavioural problems: the Child Behavior Checklist (CBCL) and the Strengths and Difficulties Questionnaire (SDQ).

**Methods:**

Item content of the CBCL and SDQ was mapped onto four dimensions: emotional problems, peer relationship problems, hyperactivity/inattention and conduct problems. A diverse test sample was drawn from four prospective longitudinal birth cohort studies in Australia and Europe who used one or both instruments. The pooled sample included 5188 data points assessing children and adolescents aged 6–13 years (*N* = 257–704 participants per cohort). Measurement invariance was assessed using latent variable multi‐group confirmatory factor analysis.

**Results:**

Fifteen items from the CBCL and SDQ were mapped onto four dimensions allowing for measurement invariance testing as part of a stepwise process. Partial strict invariance between CBCL and SDQ assessments was established for all four dimensions.

**Conclusions:**

The harmonised dimensions of emotional, peer relationship, hyperactivity/inattention and conduct problems are invariant across the CBCL and SDQ suggesting that these dimensions can be reliably compared with limited measurement error.

**Abbreviations**
ASEBA: Achenbach System of Empirically Based Assessment
BLS: Bavarian Longitudinal Study
CBCL: Child Behavior Checklist
CFA: Confirmatory factor analysis
CFI: Comparative Fit Index
EP/ELBW: Extremely preterm/extremely low birth weight
RMSEA: Root Mean Square Error of Approximation
SDQ: Strengths and Difficulties Questionnaire
SRMR: Standardised root mean square residual
VIBeS: Victorian Infant Brain Studies
VP/VLBW: Very preterm/very low birth weight

## 1 INTRODUCTION

Worldwide, up to 20% of children and young people (aged 4–19) experience mental health disorders at any given time (Barican et al., 2022; Green et al., 2005; Lawrence et al., 2015; Newlove‐Delgado et al., 2022; Polanczyk et al., 2015; Sadler et al., 2018). Mental health problems can have substantial long‐term negative effects on children and adolescents, including health and wellbeing, school success, and relationships with friends and family (Ceccarelli et al., 2022; Green et al., 2005; Lawrence et al., 2015), and can negatively affect adult functioning (Copeland et al., 2015). Childhood studies (age 4–12 years) that have used dimensional measures report that up to 38% have an emotional problem (Jaekel et al., 2018; Polanska et al., 2021) and 28% have attention/attention deficit hyperactivity disorder problems (Alemany et al., 2021; Polanska et al., 2021).

Two widely used screening instruments for mental health problems that are used for both clinical and research purposes are the Child Behavior Checklist (CBCL, Achenbach system of empirically based assessment [ASEBA]) (Achenbach et al., 2008; Achenbach & Rescorla, 2001) and the Strengths and Difficulties Questionnaire (SDQ) (Goodman, 2001). The CBCL and the SDQ are cross‐culturally valid and reliable, and have been administered across a wide range of populations (Achenbach et al., 2008; Achenbach & Rescorla, 2001; Goodman, 2001). They both have excellent diagnostic utility for psychiatric disorders in childhood (Biederman et al., 2020; Johnson et al., 2014) and provide continuous, dimensional data with an established factor structure. Both instruments have shown comparable validity within clinical and research settings (Dang et al., 2017; Klasen et al., 2000; Kovacs & Sharp, 2014). The factor structure of the CBCL (the version for ages 4–18 years) includes eight syndrome scales (anxious/depressed, withdrawn/depressed, somatic complaints, social problems, thought problems, attention problems, rule‐breaking behaviour, and aggressive behaviour) (Achenbach et al., 2008) whereas the SDQ (the version for ages 4–17 years) provides five scales (emotional problems, peer relationship problems, hyperactivity/inattention, conduct problems, and prosocial behaviour) (Goodman, 2001).

Researchers and clinicians are interested in reliable interpretation of information about children's and adolescents' mental health. It is therefore important to know and to be able to compare data about prevalence rates and the burden of disease. Large‐scale observational studies with their wealth of information on mental health outcomes provide the opportunity for pooling and cross‐referencing data, and for comparing outcomes across samples and cohorts. Meta‐analytic studies, for instance, can provide external replication and validation of previous findings (Duncan et al., 2014). Collaborative work and pooling of existing data are frequently needed to ensure that sufficient sample sizes are achieved to produce reliable results in studying subgroups, as well as identifying universal mechanisms (Duncan et al., 2014; Fortier et al., 2017; The Academy of Medical Sciences, 2015). Further, collaborations can help reduce duplications of research, obtain external replication and validation of findings, and can facilitate multidisciplinary work (Chalmers et al., 2014; Duncan et al., 2014). In order to increase research outputs and opportunities, funders not only encourage but often require researchers or research groups to plan and implement provisions for the reuse and sharing of data (Medical Research Council (MRC), 2016).

However, pooling data across studies is not straight‐forward as often different instruments are used to assess mental health outcomes. To address this problem, retrospective harmonisation can be applied to combine data from observational as well as clinical studies. Retrospective data harmonisation and the pooling of existing data provide an avenue to facilitate future collaborative work. Such pooling and analysis of existing data (Medical Research Council (MRC), 2016; Ohmann et al., 2017) allows individual participant data meta‐analysis, secondary analysis, cross‐referencing and comparing data across existing cohorts. Data pooling is also highly relevant for investigations across different assessment points within one cohort or study where the CBCL and the SDQ have been used.

In the past, retrospective data harmonisation of the ASEBA scales and SDQ has included converting scale scores into *z*‐scores (to a mean of 0 and a standard deviation of 1) (Pyhala et al., 2017), or using percentiles or predetermined cut‐offs to define mental health disorders (Alemany et al., 2021; Farkas et al., 2023). Pyhala et al. (2017) harmonised different versions of the ASEBA (i.e. the Young Adult Self Report and the Adult Self Report) where all dimensions were based on the same items. In contrast, Farkas et al. (2023) and Alemany et al. (2021) harmonised scales of the CBCL and SDQ across different cohort studies by using established instrument‐specific cut‐offs for at‐risk/problematic behaviour. This approach does not allow for the differences in the number and content of the items harmonised under the same dimension. However, harmonising items to one dimension that appears the same or similar across different measures can lead to measurement error. Measurement error pertains to differences in interpretation across groups or within the same individuals over time (e.g. across different assessment waves in longitudinal studies), and as a result arises when the meaning of the construct and how individuals interpret and respond to items may differ (Putnick & Bornstein, 2016). Measurement error can bias comparisons between studies that use different instruments which may lead to inaccuracy or misinterpretation of results (McElroy et al., 2020), especially if its effect is not accounted for in analyses.

To our knowledge, no study has attempted to harmonise the CBCL and SDQ by matching *item‐level data*. Harmonising item‐level data provides the advantage that the same or similar items are mapped to the same latent construct, ensuring a like‐for‐like approach that reflects the same content, irrespective of the source (i.e. CBCL and SDQ). Given the well‐established factor structure of the CBCL and SDQ, the present study focused on harmonising item‐level data from both instruments by mapping their items to the established dimensional factor structure of the SDQ. Importantly, this approach not only provides harmonised dimensional data but also allows for measurement invariance testing in order to estimate and limit the effect of measurement error.

Measurement equivalence or invariance testing is a psychometric method that can be employed to verify that the relationships between items and constructs are the same across instruments (i.e. CBCL vs. SDQ) (McElroy et al., 2020; Putnick & Bornstein, 2016). One of the main statistical frameworks to test measurement invariance is confirmatory factor analysis (CFA). Measurement invariance testing with CFA follows a structured, stepwise procedure where CFA model fits with increasing parameter constraints (i.e. loadings, intercepts, and residuals) are compared. Three common steps are recommended when testing whether a construct is invariant (Putnick & Bornstein, 2016): Step (1) the configural model tests whether the same measurement model is appropriate in each group; Step (2) the metric or weak factorial model tests whether the same construct is being measured across groups; and Step (3) the scalar or strong factorial model tests whether individuals interpret measures and respond in the same way. Other harmonisation approaches do not offer such statistical estimations of underlying measurement error.

The current study tested to what extent item‐level data from the CBCL and SDQ could be harmonised by mapping items from both instruments to the same mental health dimensions (i.e. emotional problems, peer relationship problems, hyperactivity/inattention, and conduct problems). Measurement invariance of the harmonised dimensions across the two instruments (i.e. CBCL and SDQ) was assessed using a stepwise process.

## 2 METHODS

### 2.1 Study sample

We utilised data from four prospective birth cohort studies that followed children born very preterm (<32 weeks' gestation or with a birth weight <1500 g) or extremely preterm (<26 weeks' gestation or with a birth weight <1000 g), alongside children born at term (≥37 weeks' gestation) as a normative reference group in three countries (Table 1): the Victorian Infant Brain Studies (VIBeS) (Treyvaud et al., 2013) cohort in Australia, born in 2001–2003; the EPICure cohort, born in 1995 in the UK and Ireland (Marlow et al., 2005); the EPICure 2 cohort, born in 2006 in England (Moore et al., 2012); and the Bavarian Longitudinal Study (BLS) cohort in Germany, born in 1985/1986 (Riegel et al., 1995). Preterm children were recruited at birth in all four studies. Term controls were recruited at birth (VIBeS and BLS) or at school‐age (EPICure and EPICure 2).

**TABLE 1:** Sample numbers and total data points across measures, informants, ages and cohorts.

Cohort	Birth year	Country	Measure	Informant	Age at assessment	*N*
VIBeS	2001–2003	Australia	SDQ	Parent	7 years	257
EPICure	1995	United Kingdom & Ireland	SDQ	Parent	6 years	372
			SDQ	Teacher	6 years	378
			SDQ	Parent	11 years	357
			SDQ	Teacher	11 years	346
EPICure 2	2006	England	SDQ	Parent	11 years	312
			SDQ	Teacher	11 years	270
			SDQ	Self‐report	11 years	310
BLS	1985/1986	Germany	CBCL	Parent	6 years	704
			CBCL	Parent	8 years	656
			SDQ	Parent	13 years	611
			SDQ	Self‐report	13 years	615
Total data points			CBCL			1360
			SDQ			3828
			CBCL & SDQ combined			5188

Abbreviations: CBCL, Child Behavior Checklist; SDQ, Strengths and Difficulties Questionnaire.

### 2.2 Measures

All four studies collected CBCL and/or SDQ data from multiple informants (parents, teachers, and child/adolescent self‐report) and across different ages ranging from 6 to 13 years of age (Table 1). Both measures provide continuous scale level data and have a similar question structure, and ordinal response format.

#### 2.2.1 The CBCL (version for ages 4–18 years; ASEBA scales)

The ASEBA scales offer age‐appropriate instruments for children and adolescents including the CBCL (parents as informants) (Achenbach & Rescorla, 2001). ASEBA versions for teachers (Teacher's Report Form) and self‐report (Youth Self‐Report) are also available. The CBCL assesses behavioural/mental health problems with 112 items on a 3‐point Likert‐type scale (0 = not true, 1 = somewhat true, 2 = often true) (Achenbach et al., 2008). Some country‐specific norms (e.g. (Remschmidt & Walter, 1990), and language translations are available and the cross‐cultural validity of the CBCL has been confirmed (Achenbach, 2019; Achenbach et al., 2008)).

#### 2.2.2 The SDQ (version for ages 4–17 years)

The SDQ is a screening instrument containing 25 items scored on a 3‐point Likert‐type scale (0 = not true, 1 = somewhat true, 2 = certainly true) (Goodman, 1997). SDQ versions are available for parents, teachers, and self‐report, including multiple language translations. SDQ factor structure and factorial validity have been supported by various studies (Goodman, 2001) and across ethnic groups (Zwirs et al., 2011).

### 2.3 Procedure and statistical analysis

#### 2.3.1 Mapping item‐level SDQ and CBCL data onto dimensions

**FIGURE 1:**
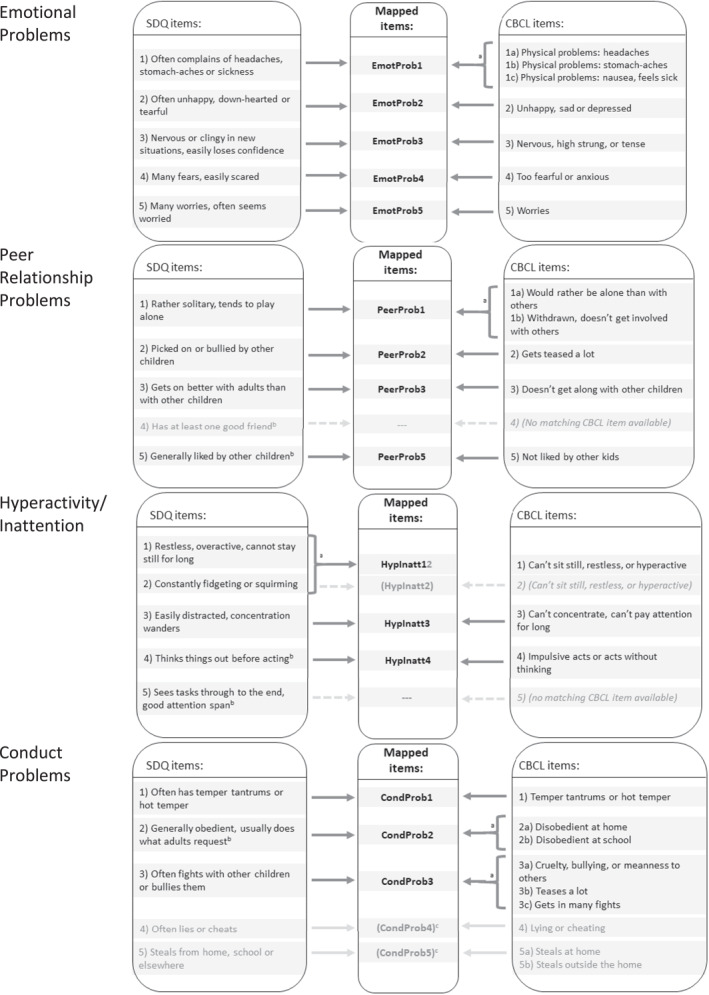
Theory and content‐based mapping of SDQ and CBCL item‐level data to four dimensions. ^a^If multiple CBCL or SDQ items could be matched to one mapped item/variable, the items were combined and the mean was calculated. ^b^Reverse coded. ^c^These items were excluded from the multi‐group confirmatory factor analyses as neither full nor partial measurement invariance could be demonstrated. CBCL, Child Behavior Checklist; SDQ, Strengths and Difficulties Questionnaire.

Individual item content of both instruments was jointly mapped to four dimensions by two authors (NB, JJ) through an iterative process, following the dimensional structure of the shorter SDQ: (1) emotional problems, (2) peer relationship problems, (3) hyperactivity/inattention, and (4) conduct problems (Figure 1). The method of mapping items from the CBCL to items and dimensions of the shorter SDQ was chosen in order to use the largest common denominator between both instruments.

Specifically, across all four data sets, item level data of the CBCL and the SDQ were recoded into one mapped variable. For instance, the three CBCL items, ‘physical problems: headaches’, ‘physical problems: stomach‐aches’ and ‘physical problems: nausea, feels sick’ were identified to match the first SDQ item ‘often complains of headaches, stomach‐aches or sickness’. Accordingly, the single SDQ item was simply recoded into the mapped variable ‘EmotProb1’, whereas the three matched CBCL items were first combined and averaged, resulting in one mapped variable ‘EmotProb1’ that reflected the largest common denominator and directly corresponded to the SDQ item (Figure 1).

In order to test invariance of item‐level data, all data sets including data points from all cohorts (VIBeS, EPICure, EPICure2 and BLS) of the mapped items from both instruments (SDQ and CBCL) across all informants (parents, teachers, self‐report) and all ages (6, 7, 8, 11 and 13 years) were pooled into one data set. Specifically, the data were structured so that one row represented one assessment, irrespective of the instrument (SDQ and CBCL) used, the informant and age at assessment.

#### 2.3.2 Measurement invariance testing

**FIGURE 2:**
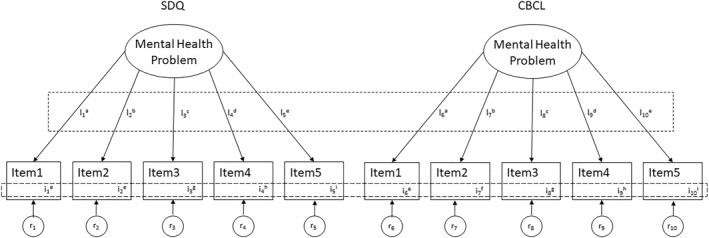
Multi‐group latent variable confirmatory factor analysis: example model (adapted from McElroy et al., 2020; Putnick & Bornstein, 2016). CBCL, Child Behavior Checklist; SDQ, Strengths and Difficulties Questionnaire; i, item intercepts; l, item loadings; r, item residuals.

Following published guidelines, measurement invariance was tested using latent variable multi‐group (group 1 = SDQ vs. group 2 = CBCL) CFA (Figure 2) (McElroy et al., 2020; Putnick & Bornstein, 2016). Invariance was established separately for each of the four dimensions: emotional problems, peer relationship problems, hyperactivity/inattention and conduct problems. A structured, stepwise procedure was followed, where CFA model fit with increasing parameter (i.e. item loadings/weights and item intercepts/means) constraints were compared for models nested within each other, that is, the configural, metric (or weak factorial), and scalar (or strong factorial) model (Putnick & Bornstein, 2016). In addition, a further step in this measurement invariance testing framework has been described: the residual model tests for residual invariance (or strict or invariant uniqueness). This model tests whether the sum of the variance of items not shared with the factor and error variance is similar across groups. However, as residual invariance has no effect on the interpretation of latent mean differences, most studies omit this step (Putnick & Bornstein, 2016). Accordingly, residual invariance was not tested.

Model fits were based on three model fit indices: the root mean square error of approximation (RMSEA), comparative fit index (CFI), and standardised root mean square residual (SRMR). Acceptable model fits were defined as: RMSEA ≤ 0.08; CFI ≥ 0.90; SRMR ≤ 0.08 (Brown, 2015; Little & Card, 2013). Measurement invariance was established if the change of model fit values within the nested models was not substantial, that is, remained within the following thresholds: ΔRMSEA ≤ 0.015; ΔCFI ≤ 0.010; ΔSRMR ≤ 0.035 (Putnick & Bornstein, 2016).

In the first step, the *configural model* served as the baseline or reference model and tested whether the same measurement assumptions applied across groups. In this model, all parameters (item loadings and intercepts) were allowed to vary freely. The model fits of the subsequent models (i.e. the metric and scalar models) were compared with the model fits of this baseline model.

In the second step, the *metric model* tested whether the same construct was being measured across groups (CBCL and SDQ). That is, this model tested whether the associations between indicator items and the latent factor were consistent across groups. Therefore, all item loadings were held constrained or invariant in this model. If the overall model fits for the metric model were not substantially worse compared to model fits of the configural model, metric invariance was supported.

In the third and final step, the *scalar model* kept all item loadings and item intercepts constrained. If the overall model fits did not change substantially compared to the model fits of the configural model, it meant that participants interpreted the responses in the same way across groups and scalar invariance was supported.

Previous studies have shown that both full metric and scalar invariance are often not achieved (Murray et al., 2022; Putnick & Bornstein, 2016; Van Lieshout et al., 2015). As a result, the accepted alternative strategy is to assess partial measurement invariance instead. As such, if full metric or scalar measurement invariance could not be achieved in the present study, modification indices and expected parameter changes were reviewed. Guided by these indices, parameters (i.e. loadings or intercepts, or both) were released until acceptable model fits and measurement invariance were achieved (Putnick & Bornstein, 2016).

## 3 RESULTS

### 3.1 Study sample

Pooling all data sets yielded 5188 data points across both measures (CBCL: 1360 data points; SDQ: 3828 data points), and across cohorts, informants, and ages (Table 1).

### 3.2 Mapping of item‐level data

Items of the CBCL and SDQ were mapped onto the same four dimensions following the process described above: emotional problems, peer relationship problems, hyperactivity/inattention, and conduct problems (Figure 1).

### 3.3 Measurement invariance of the harmonised dimensions

The results of the measurement invariance tests across the two groups (CBCL and SDQ) are presented in Table 2.

**TABLE 2:** Assessment of measurement invariance for the four harmonised scales.

Model	Data points	Chi‐square (DF)	RMSEA	CFI	SRMR	ΔRMSEA	ΔCFI	ΔSRMR	Invariance
Emotional problems (5 items)									
Configural	5108	156.018 (10)	0.076	0.965	0.029				
Metric		196.480 (14)	0.071	0.957	0.046	0.005	0.008	−0.017	Yes
Scalar		497.035 (18)	0.102	0.886	0.058	−0.026	0.079	0.058	No
Partial scalar		203.174 (16)	0.068	0.956	0.047	0.008	0.009	−0.018	Yes
Peer relationship problems (4 items)									
Configural	5134	47.579 (4)	0.065	0.978	0.022				
Metric		185.750 (7)	0.100	0.908	0.079	−0.035	0.070	−0.057	No
Partial metric		47.583 (4)	0.065	0.978	0.022	0.000	0.000	0.000	Yes
Scalar		303.488 (2)	0.148	0.798	0.077	−0.083	0.180	−0.055	No
Partial scalar		15.361 (1)	0.075	0.985	0.026	−0.010	−0.007	−0.004	Yes
Hyperactivity/Inattention (3 items)									
Configural	5117	8.522 (1)	0.054	0.998	0.018				
Metric		47.610 (2)	0.094	0.986	0.059	−0.040	0.012	−0.041	No
Partial metric		10.202 (1)	0.060	0.997	0.027	−0.006	0.001	−0.009	Yes
Scalar		62.383 (3)	0.088	0.982	0.050	−0.034	0.016	−0.032	No
Partial scalar		10.646 (2)	0.061	0.997	0.028	−0.007	0.001	−0.010	Yes
Conduct problems (3 items)									
Configural	5153	1.828 (1)	0.018	0.999	0.006				
Metric		151.891 (2)	0.171	0.833	0.093	−0.153	0.166	−0.087	No
Partial metric		1.828 (1)	0.018	0.999	0.006	0.000	0.000	0.000	Yes
Scalar		84.332 (3)	0.103	0.910	0.056	−0.085	0.089	−0.050	No
Partial scalar		6.913 (2)	0.031	0.995	0.014	−0.013	0.004	−0.008	Yes

*Note*: The grey values indicate that the change of model fit values were outside the thresholds for measurement invariance (ΔRMSEA ≤ 0.015; ΔCFI ≤ 0.010; ΔSRMR ≤ 0.035).

Abbreviations: CFI, comparative fit index; RMSEA, root mean square error of approximation; SRMR, standardised root mean square residual.

#### 3.3.1 Emotional problems (5 harmonised items)

**FIGURE 3:**
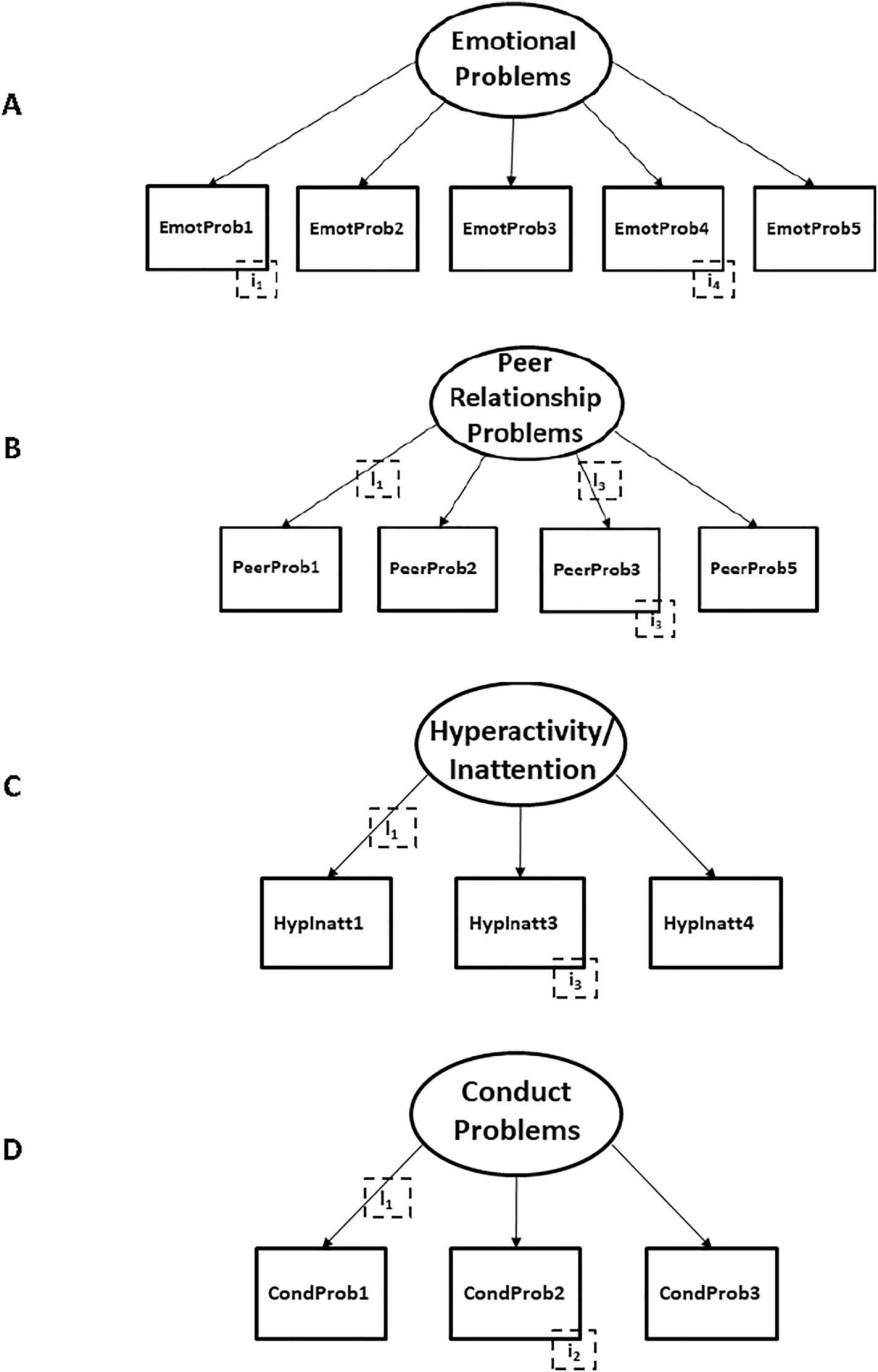
Mapped models of latent variable multi‐group confirmatory factor analyses (statistical parameters not shown for simplicity; freed item loadings [l] and item intercepts [i] are indicated). (a) EmotProb1–5, (b) PeerProb1–5, (c) HypInatt1–4 and (d) CondProb1–3 indicate harmonised items in the respective model.

The configural model for emotional problems had acceptable fit values (RMSEA = 0.076, CFI = 0.965, SRMR = 0.029). The model fit of the metric model (i.e. constrained item loadings) remained within recommended cut‐offs for nested models (ΔRMSEA ≤ 0.015, ΔCFI ≤ 0.010, ΔSRMR ≤ 0.035). However, after additionally constraining the item intercepts in the scalar model, model fit worsened substantially (ΔRMSEA > 0.015, ΔCFI > 0.010, ΔSRMR > 0.035). Therefore, modification indices were reviewed and intercepts of two items freed (EmotProb1 and EmotProb4). As a result, model fit improved and partial scalar invariance was established (Figure 3a).

#### 3.3.2 Peer relationship problems (4 harmonised items)

The fit values for the configural model were acceptable (RMSEA = 0.065, CFI = 0.978, SRMR = 0.022). After constraining the item loadings for the metric model, fit values worsened substantially (ΔRMSEA > 0.015, ΔCFI > 0.010, ΔSRMR > 0.035). Guided by modification indices, loadings for items PeerProb1 and PeerProb3 were released to improve model fit and to achieve partial metric invariance. Introducing constraints to the item intercepts led to a worsening of the scalar model fit (ΔRMSEA > 0.015, ΔCFI > 0.010, ΔSRMR > 0.035). But freeing the intercept of item PeerProb3 resulted in partial scalar invariance (Figure 3b).

#### 3.3.3 Hyperactivity/inattention (3 harmonised items)

The configural model had acceptable fit values (RMSEA = 0.054, CFI = 0.998, SRMR = 0.018). However, once item loadings were constrained, the fit values for the metric model were outside recommended cut‐offs (ΔRMSEA > 0.015, ΔCFI > 0.010, ΔSRMR > 0.035). Partial metric invariance was obtained by releasing the loading for item HypInatt1. After restricting item intercepts, partial scalar invariance was rejected initially. However, partial scalar invariance was established by releasing the intercept of item HypInatt3 (Figure 3c).

#### 3.3.4 Conduct problems (3 harmonised items)

The model fits for the configural model including all five harmonised items for the construct ‘conduct problems’ were acceptable (RMSEA = 0.074, CFI = 0.933, SRMR = 0.035). Measurement invariance for the metric model could not be obtained but after releasing the loadings for the two harmonised items 1 (‘temper tantrums or hot temper’) and 5 (‘steals from home, school or elsewhere’) partial metric invariance was established. However, neither full nor partial scalar invariance could be obtained for the 5‐item model.

Given the failure to establish measurement invariance and the fit values for the configural model for the 5‐item model, we considered a 3‐item model, omitting the items ‘lying or cheating’ and ‘steals from home, school or elsewhere’. The fit values for this configural model were excellent (RMSEA = 0.019, CFI = 0.999, SRMR = 0.007). Although both full metric and scalar models were not achieved, partial metric invariance was established by freeing the loading of item CondProb1 and partial scalar invariance by freeing the intercept of item CondProb2 (Figure 3d).

## 4 DISCUSSION

This study demonstrated partial measurement invariance for the four dimensions of emotional problems, peer relationship problems, hyperactivity/inattention and conduct problems, based on harmonised item‐level data from the CBCL and the SDQ in a sample of children and adolescents born in four countries and across two decades. For the first time, this provides proof‐of‐concept that the CBCL and SDQ can be harmonised with limited measurement error. Measurement invariance testing via latent factor multi‐group CFA is an important and reliable psychometric procedure that allows testing of a psychological concept across measures and groups. Once measurement invariance is achieved valid comparisons of differences or relations based on latent factors can be made, while the limitation of measurement error is removed or alleviated. Hence, establishing measurement invariance allows for a reliable assessment of the prevalence of mental health problems across groups (Putnick & Bornstein, 2016).

It is important to note that although measurement error was limited in the present study, some element of bias in the estimates may still be present. This element of bias is unquantifiable and may be attributed to the released parameters (i.e. item loadings and item constraints). In keeping with existing guidelines, at least half of these parameters should be invariant across groups in order to be able to use the latent factor in meaningful analyses and comparisons (McElroy et al., 2020; Putnick & Bornstein, 2016), which was achieved in the current study. In addition, it is noteworthy that some level of non‐invariance across different groups or measures is usually expected. Children's mental health changes over time and age, in particular with regard to age of onset and symptomology of psychopathological concepts, including emotional problems, peer relationship problems, hyperactivity/inattention, and conduct problems (American Psychiatric Association, 2013; World Health Organization, 2022). For instance, in children, emotional problems may be expressed as somatic problems, such as headache or abdominal pain, while in adolescents emotional problems may manifest as irritability (World Health Organization, 2022). Furthermore, it is noteworthy that the sample of the current study is heterogeneous. That is, data points are included from different age ranges (middle childhood and early adolescence: age 6–8 and age 11–13 years, respectively) and informants (parent‐report, self‐report and teacher‐report) across both measures (SDQ and CBCL). While a selection of more homogeneous samples may provide superior model fits and invariance across instruments, our goal here was to document that harmonisation and invariance can be achieved despite substantial heterogeneity.

Importantly, the findings of the current study provide methodological insights that are not restricted to one research area or topic. That is, retrospective item‐level data harmonisation as described here can be applied to any investigation that has used the CBCL, or possibly other ASEBA scales, and the SDQ. To encourage and facilitate future collaborative work and data sharing across research groups it is vital to share methodologies and scripts of how to harmonise item‐level data that have been collected with different mental health screening instruments.

Our findings further promote item‐level harmonisation. Item‐level harmonisation provides more granular data than scale‐level harmonisation and ensures that conceptually equivalent data are combined across instruments. For instance, scale‐level harmonisation neglects measurement error, whereas our approach minimises this error. Despite differences, the SDQ and the CBCL partially overlap in their content and include similar items and response categories. This makes the two scales highly suitable for item‐level harmonisation. Other instruments that assess mental health and behaviour in children and adolescents and that are used frequently, include similar items as the SDQ and/or the CBCL (e.g. the Rutter Scale (Rutter et al., 1970) or the Behavior Rating Inventory of Executive Function (Gioia et al., 2000)) Despite differences in response categories across these instruments, it may be possible to not only harmonise items according to their content but also to harmonise response categories, as shown in a recent study of data from six British Cohorts (McElroy et al., 2020). Future research should extend item‐level data harmonisation and measurement invariance across other widely used dimensional screening instruments for mental health. If measurement invariance can be achieved across multiple mental health screening instruments, additional cohort data can be added and extended to item‐level harmonisation for instruments that assess other outcomes, for example, health‐related quality of life, or life satisfaction. Finally, future research should investigate1the predictive and discriminative validity of the newly harmonised scales of the current study compared to unharmonised single‐instrument studies that have used either the SDQ or the CBCL.

Overall, the findings of the current study are important forresearchers and clinicians interested in assessing children's and adolescents' mental health. Based on the results of the current study, future cross study investigations of mental health should consider harmonising data at the item level rather than at the scale level.

## 5 CONCLUSIONS

Harmonisation of item‐level data from the SDQ and the CBCL into dimensions of emotional problems, peer relationship problems, hyperactivity/inattention and conduct problems was achieved as described in Figure 1. Congruency of meaning and underlying conceptualisation of these newly harmonised dimensions was demonstrated. These findings provide the opportunity for pooling and retrospective data harmonisation across a variety of samples and research topics within national and international collaborations.

## AUTHOR CONTRIBUTIONS

**Nicole Baumann**: Conceptualization; data curation; formal analysis; funding acquisition; investigation; methodology; project administration; validation; visualization; writing – original draft; writing – review & editing. **Peter J. Anderson**: Conceptualization; investigation; supervision; validation; writing – review & editing. **Samantha Johnson**: Conceptualization; funding acquisition; investigation; supervision; validation; writing – review & editing. **Neil Marlow**: Investigation; validation; writing – review & editing. **Dieter Wolke**: Investigation; validation; writing – review & editing. **Julia Jaekel**: Conceptualization; funding acquisition; investigation; methodology; supervision; validation; writing – review & editing.

## CONFLICT OF INTEREST STATEMENT

The authors declare no conflicts of interest.

## ETHICS STATEMENT

All cohort studies had received country‐specific ethical reviews, with parents providing written informed consent, and all adhered to the Declaration of Helsinki.

## Data Availability

The data for this study are not publicly available due to health data protection. However, scripts for conducting item‐level data harmonisation described in this study are available on request from the corresponding author.
